# Oral health among refugees and asylum seekers utilizing Médecins du Monde clinics in mainland Greece, 2016–2017

**DOI:** 10.1186/s12903-024-04841-2

**Published:** 2024-09-06

**Authors:** Sarah Elizabeth Scales, Bhumi Vora, Kyle Loftus

**Affiliations:** 1 Water, Climate, and Health Program, College of Public Health, University of Nebraska Medical Center, Omaha, NE 68198 USA; 2 Epidemiology Program, College of Health Sciences, University of Delaware, Newark, DE 19713 USA; 3 Lincoln Medical and Mental Hospital, Bronx, NY 10451 USA

**Keywords:** Oral health, Displacement, Complex emergencies, Disasters, Humanitarian health

## Abstract

**Background:**

The oral health of refugees and asylum seekers is understudied. However, oral health has important implications for overall health and wellbeing. This study addresses this gap by characterizing oral health care utilization in Médecins du Monde (MdM) clinics across mainland Greece from 2016 – 2017.

**Methods:**

A retrospective cross-sectional study design was used to estimate proportional morbidities for caries, extraction, developmental, periodontal disease, preventive, and other oral health outcomes. The association between physical health conditions and consultations of interest – upper respiratory tract infections (URTIs) and reproductive health consultations – and oral health were compared using odds ratios (OR) and 95% confidence intervals (CIs). Oral health outcomes between Afghans and Syrians were compared using odds ratios and 95% CIs.

**Results:**

Caries (39.44%) and extractions (28.99%) were highly prevalent in our study population. The utilization of preventive dental consultations (37.10%) was high, particularly among males. Individuals with at least one upper respiratory tract infection (OR = 1.52; 95% CI: 1.30 – 1.77; Or = 1.90; 95% CI: 1.53 – 2.36) and women and girls with reproductive health consultations (OR = 1.30; 95% CI: 1.03 – 1.66; OR = 2.03; 95% CI: 1.49 – 2.76) were more likely to have any dental or caries specific consultations. The observed patterns in oral health needs differed between Afghans and Syrians, with Afghans more likely to have preventive screenings and less likely to have caries, extractions, or other conditions.

**Conclusions:**

Displaced populations utilizing MdM dental clinics had high levels of oral health needs, particularly for caries and extractions. The connection between oral and overall health was seen in the study population, and these findings reinforce the public health importance of oral health for improving health and wellbeing of displaced populations. Evidence-informed policy, practice, and programming inclusive of oral health are needed to address both oral and overall health of refugees and asylum seekers in Greece. Future research should investigate not only oral health care needs but also knowledge and beliefs that inform utilization patterns among displaced populations.

**Supplementary Information:**

The online version contains supplementary material available at 10.1186/s12903-024-04841-2.

## Background

Oral health is a critical indicator of overall health and is involved in many chronic diseases and systemic conditions. The Center for Disease Control and Prevention (CDC) highlights the link between poor oral hygiene and chronic diseases such as diabetes and heart disease, emphasizing that the bacterial ecosystem of the mouth can affect the entire body [1]. This connection is particularly concerning for vulnerable populations such as refugees and asylum seekers, whose oral health care needs are often unmet and understudied, indicating a gap to be addressed by public health research and policy [2]. Further, there is a high level of variability in access to primary and preventative care for refugees depending on their sending country, transit routes utilized, available resources, and other circumstantial factors [3]. Allostatic load – the cumulative effect of generational and experienced stressors – can negatively impact oral health [4], emphasizing another dimension of concern for refugees and asylum seekers. Given the interdependency of physical and oral health, it is critical to better understand and address these needs among refugees and asylum seekers, particularly as insecurity and subsequent displacement exacerbate already tenuous systems.

The relationship between dental health and overall physical health is increasingly recognized in medical and public health research [5]. Poor oral health, often a result of inadequate hygiene practices and unhealthy behaviors (e.g., tobacco and alcohol use, high sugar consumption, and nutritionally insufficient diets) can exacerbate poor physical health conditions. Conditions such as endocarditis, cardiovascular disease, complications in pregnancy and birth, and pneumonia have been linked to poor oral health [6]. Other conditions, like diabetes [7] and HIV/AIDS [8] can intensify oral health issues. In children, oral health can affect their growth, development, and general well-being [9]. There is also a growing literature base explaining the relationship between oral microbiota, respiratory infections, and inflammatory respiratory conditions [5].

This complex and bidirectional relationship underscores the need for integrated health approaches that consider oral health as a vital component of overall physical health, in both adults and children. The dental health of refugees and asylum seekers, particularly in Europe, remains an underexplored area of public health. There is little information about dental health or overall health status and healthcare access for displaced populations across Europe [10]. A scoping review by Rad et al., (2023), included 26 studies covering dental caries and periodontal diseases among refugees and asylum seekers from 2011 to 2022 [11]. The prevalences of caries in refugee communities across study sites and countries of origin were high [11], including a study in Greece where dental concerns were the most prevalent health-related problem [12]. In a review of services for refugees and asylum seekers in the United Kingdom, Fennell-Wells and Yusuf (2020) noted the scarcity of preventative screenings and routine dental care [13]. Despite isolated evidence, such as the high prevalence of dental decay among a cohort of 75 unaccompanied minors receiving care in Kent, England [14], Fennell-Wells and Yusuf also identified the lack of data characterizing the burden of oral concerns and related needs [13].

Although the oral health of refugees and asylum seekers is a critical aspect of their overall well-being, it often receives limited attention in research, health policy, and humanitarian programs [2]. This oversight can lead to significant unmet dental needs and contribute to broader health disparities among displaced populations. Our study addresses the literature gap in oral health of displaced populations, particularly in southern Europe, by characterizing utilization and reasons for consultations from dental clinics operated by Médecins du Monde (MdM) – a non-governmental, humanitarian organization – across 12 mainland Greek camps and reception centers from 2016 through 2017, following the closure of the Balkan Route and signing of the European Union-Türkiye Deal in 2016. Changes in freedom of movement from Greece toward western Europe complicated an already dire humanitarian situation in the Eastern Mediterranean. To address the vast needs of refugees and asylum seekers, MdM clinics were operationalized throughout the mainland to deliver primary, dental, mental, and sexual and reproductive healthcare.

## Methods

### Study description and population

We conducted a retrospective cross-sectional study to characterize the oral health needs and dental clinic utilization of refugees and asylum seekers with consultations at 12 Médecins du Monde (MdM) clinics operating in camps and reception centers across mainland Greece from 2016 through 2017. MdM is an implementing non-governmental organization that facilitates and provides general, dental, mental, and sexual and reproductive health care in complex emergencies across 30 countries [15]. Dental clinics were available to all across clinics operating in the 12 camps and reception centers. Datasets were developed for 1) dental data only and 2) combined primary care and dental data. We obtained these data from the Centre for Research on the Epidemiology of Disasters, Association pour l’Etude Epidemiologique des Désastres (CRED-ASED).

### Measures

We used clinical diagnoses, treatment notes, and prescribing notes to construct 6 categories of consultations, including caries, extraction, periodontal disease, developmental/eruption concerns (e.g., eruption and eruption related pain, impacted teeth, supernumerary mesiodens, and over-retained primary teeth), prevention, and other conditions (yes/no). Because of the data structure, there was significant cleaning and content analysis for appropriately categorizing data for analysis. For example, an individual could have a consultation with a diagnosis of caries and a subsequent consultation for extraction, each of which was accounted for in analyses. Similarly, an individual could have a diagnosis of caries with a treatment note of ‘extraction’ for a single clinic visit; both caries and extraction would be recorded for this individual, as well. If a single patient had multiple visits for the same condition, that individual was a assigned a ‘yes’ for the given condition.

Data were reported as clinical line-list for each visit; data on full oral health status and history were not reported in datasets used in this study. Any diagnosis that did not fit into one of the five specific categories (e.g., non-descript pain, injuries not necessitating extraction, removal of stitches) was included in the “other” category. These categories were modeled after Keboa et al., (2016), and refined for the scope of our data and purposes of the study. Two authors (SES and BV) independently developed codes, and differences (15%) were reconciled by a third author specialized in dental diagnostics (KL) [2]. We merged dental diagnoses data with primary care outcomes, including binary classifications of non-insulin dependent diabetes mellitus (NIDDM), insulin dependent diabetes mellitus, hypertension, upper respiratory infections (URTIs), and lower respiratory infections.

Demographic characteristics, including sex, age, and country of origin were reported. We categorized sex (male/female) and age (0–14; 15–49; >  = 50) as described by the United Nations Office for Disaster Risk Reduction (UNDRR) guidelines on reporting sex, age, and disability disaggregated data (SADDD) [16]. Country of origin was categorized into four groups – Syria, Afghanistan, Iraq and Kurdistan, and others, which included Algeria, Egypt, Iran, Kuwait, Lebanon, Libya, Morocco, Pakistan, Occupied Palestine, and Somalia. No information was available on duration in camp, education level, languages spoken, or other demographic characteristics.

### Analysis

We conducted data cleaning and analysis using SAS Studio OnDemand (Cary, NC). Chi-square p-values are reported for categorical variables, and median and interquartile range (IQR) are also reported for age. We report proportional morbidities (PM) and 95% confidence intervals (CIs), describing the prevalence of given conditions among dental consultations, and odds ratios (OR) and 95% CIs, describing the relationship between dental care (e.g., any dental consultations for complaint, procedure, or prevention) and physical health conditions and consultations of interest (e.g., upper respiratory tract infections and reproductive health consultations broadly). We also report odds ratios and 95% CIs comparing dental consultations among all Afghans and Syrians utilizing primary care clinics across the 12 camps.

### Ethics statement

This study, methods, and related materials were reviewed by the University of Delaware’s Institutional Review Board (IRB 2002123) and was determined to be exempt under Category 2 of the US Department of Health and Human Services regulations for the protection of human subjects in research at 45CFR 46. Secondary, deidentified data are used in this study, and, therefore, oral or written consent was not required.

## Results

### Study population

Of the 7245 individuals with primary care consultations (e.g., any cause consultation at a primary care clinic), 47.05% were from Syria, and 42.02% were from Afghanistan. Roughly 8% were from Iraq and Kurdistan, and the remaining 3% were from other countries of origin. Across all age groups, males had more primary care consultations than females. However, nearly 70% of all consultations were for women and children. Most consultations across camps were for individuals 15 – 49 years (54.22%); only 5.27% of consultations were for individuals 50 years and older. The median and mean age for primary care consultations were 18 years (IQR 23) and 20.80 $\pm$ 15.48 years, respectively.

There were 1283 individuals with dental consultations in 12 camps across Epirus, Central Macedonia, Thessaly, and Attica administrative regions of Greece. Among dental consultations, most were for Syrians (46.22%), followed closely by Afghans (43.18%). Iraqis and Kurds were just under 10% of consultations, with the remaining 1% from other countries of origin. Nearly half of dental consultations were for patients 15 to 49 years of age (49.26%); the mean age of dental patients was 20.5 $\pm$ 14.2 years and the median age was 16 years (IQR 21). Males (52.77%) had more dental consultations than females (47.23%). Descriptive statistics for dental consultations only and for primary care consultations are summarized in Table 1.

**Table 1:** Frequencies of dental consultations only and both dental and primary care consultations

		Dental Consultations Only			Both Dental and Primary Care Consultations		
		N	%	χ2 *p*-value	N	%	χ2 *p*-value
Countries of Origin	Afghanistan	554	43.18%	< 0.0001	3044	42.02%	< 0.0001
	Iraq	123	9.59%		571	7.88%	
	Other Countries	13	1.01%		221	3.04%	
	Syria	593	46.22%		3409	47.05%	
Age Groups	0–14	606	47.23%	< 0.0001	2935	40.51%	< 0.0001
	15–49	632	49.26%		3926	54.22%	
	> = 50	45	3.51%		382	5.27%	
Sex	Female	606	47.23%	< 0.0001	3371	46.53%	0.0475
	Male	677	52.77%		3874	53.47%	
Camps	Elliniko	335	26.11%	< 0.0001	1841	25.41%	< 0.0001
	Malakasa	164	12.78%		1130	15.60%	
	Kavala	20	1.56%		342	4.72%	
	Raidestos	162	12.63%		1010	13.94%	
	Doliana	80	6.24%		139	1.92%	
	Faneromeni—Lakkas	101	7.87%		284	3.92%	
	Filipiada	133	10.37%		399	5.51%	
	Katsikas	153	11.93%		614	8.47%	
	Konitsa	14	1.09%		160	2.21%	
	Koutsochero	42	3.27%		847	11.83%	
	Trikala	57	4.44%		360	4.97%	
	Volos	22	1.71%		109	1.50%	

### Dental consultation patterns

**Fig. 1:**
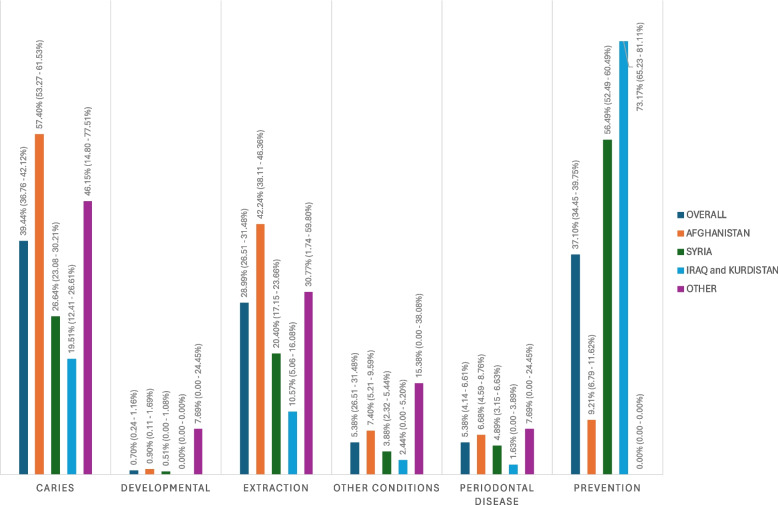
Overall and country-specific proportional morbidities and 95% confidence intervals for categories of reasons for consultation

PMs for reasons for consultation by country of origin are summarized in Fig. 1. Caries had the highest PM (39.44%; 95% CI: 36.76 – 42.12%), followed by prevention-related concerns (37.10%; 95% CI: 34.45 – 39.75%). Consultations for developmental and eruption-related concerns had the lowest PM across countries of origin. For all countries of origin, the prevalence of periodontal disease was less than 8.00%, and the overall PM was 5.38% (95% CI: 4.14 – 6.61%). The PMs of caries and extractions were highest for Afghans, with 57.40% (95% CI: 53.27 – 61.53%) and 42.24% (95% CI: 38.11 – 46.36%) of individuals with dental consultations having caries or needing dental extractions, respectively. Preventative consultations had the highest PM for Syrians (PM = 56.49%; 95% CI: 52.49 – 60.49%) and Iraqis and Kurds (PM = 73.17%; 95% CI: 65.23 – 81.11%).

Age- and sex-specific consultation patterns are summarized in Table 2. Preventive screenings were the most common consultation type (72.61%) followed by caries (17.66%) and extractions (16.50%) for children (0–14). Extraction and caries constituted the highest proportional morbidities for adults (15 – 49 years) and older adults (< = 50 years). Proportional morbidities were not significantly different between females and males. Women were 15% less likely to have preventive screenings compared to males (OR = 0.85; 95% CI: 0.68 – 1.07%; not shown).

**Table 2:** Proportional morbidities and 95% confidence intervals for consultation reason within Sex, Age, and Disability Disaggregated Data (SADDD)-defined age and sex groups

	Age						Sex			
	0–14 years		15—49		> = 50		Female		Male	
Caries	17.66%	(14.61—20.70%)	38.92%	(35.11—42.74%)	28.89%	(15.12—42.66%)	41.09%	(37.16—45.02%)	37.96%	(34.30—41.63%)
Developmental	0.66%	(0.01—1.31%)	0.79%	(0.10—1.48%)	0.00%	(0.00—0.00%)	0.50%	(0.00—1.06%)	0.89%	(0.18—1.59%)
Extraction	16.50%	(13.54—19.47%)	39.24%	(35.42—43.06%)	53.33%	(38.18—68.49%)	28.38%	(24.78—31.98%)	29.54%	(26.10—32.99%)
Other	2.15%	(0.99—3.30%)	8.22%	(6.08—10.38%)	8.89%	(0.24—17.54%)	6.44%	(4.48—8.39%)	4.43%	(2.88—5.99%)
Periodontal Disease	0.33%	(0.00—0.79%)	9.97%	(7.63—12.31%)	8.89%	(0.24—17.54%)	5.94%	(4.05—7.83%)	4.87%	(3.25—6.50%)
Prevention	72.61%	(69.05—76.17%)	5.06%	(3.35—6.78%)	8.89%	(0.24—17.54%)	35.15%	(31.34—38.96%)	38.85%	(35.17—42.53%)

### Dental consultations and physical health conditions and consultations of interest

Those with at least one upper respiratory tract infection diagnosis had 1.52 times higher odds of any diagnosed dental problem (95% CI: 1.30 – 1.77) and nearly twice the odds of caries (OR = 1.90; 95% CI: 1.49 – 2.76). Among only women, those with at least one reproductive health consultation – including but not limited to antenatal care, family planning and contraception, and menstruation-related concerns – were 1.30 times more likely to have any dental consultation compared to those without a reproductive health consultation (OR = 1.30; 95% CI: 1.03 – 1.66). Women with reproductive health consultations were twice as likely to have a caries diagnosis compared to those without reproductive health consultations (OR = 2.03; 95% CI: 1.49 – 2.76). These findings are summarized in Table 3.

**Table 3:** Dental consultations and comorbid conditions of interest (*n* = 7245)

Physical Health Conditions and Consultations	Any Oral Health Problem		Caries	
	OR	95% CI	OR	95% CI
Upper Respiratory Infections	1.52	(1.30—1.77)	1.90	(1.53—2.36)
Reproductive Health Consultations^1^	1.30	(1.03—1.66)	2.03	(1.49—2.76)

^1^Reproductive health consultations are restricted to women and girls of reproductive age

### Comparing dental consultations among Afghans and Syrians (*n* = 6453)

Afghans were significantly less likely to have caries (OR = 0.48; 95% CI: 0.38 – 0.60), other conditions (OR = 0.52; 95% CI: 0.29 – 0.92), or diagnoses necessitating extraction (OR = 0.54; 95% CI: 0.41 – 0.73) when compared to Syrians. They were 3.8 times more likely to have preventative consults compared to Syrians (OR = 3.79; 95% CI: 2.52 – 5.71). There was no appreciable difference in odds of consultations for periodontal disease or developmental concerns between the two nationalities. These data are summarized in Supplementary File 1.

## Discussion

Most studies of oral health for displaced persons from Afghanistan, Syria, and Iraq have been conducted in Eastern Mediterranean Region countries, including but not limited to Lebanon, Syria, Jordan, Occupied Palestine, Morocco, Iraq, and Afghanistan [11]. However, there have been few studies in European countries receiving refugees and asylum seekers via the Eastern Mediterranean transit routes. A systematic review, Keboa et al., (2016), found only one study on oral health among Eastern Mediterranean refugees and asylum seekers in southern Europe from 1990 to 2014 [2]. A scoping review which included literature through 2020 similarly found only one study addressing oral health in southern Europe [17]. Fennell-Wells and Yusuf reported on two studies in Greece, one focusing on oral health and the other including oral health as part of primary care.

Dental consultation patterns.

Among refugee and asylum seekers who were moved from Greek island to mainland camps between 2015 and 2016, dental clinic needs broadly, and caries specifically, increased, though not significantly, once they accessed care on the mainland [18]. This could be driven by various considerations, including more access to care in Athens-Piraeus Port or limited access to and increased duration of disruptions to health and hygiene-promoting facilities and resources. The high prevalence of caries and related extractions are reflected in our findings. Additionally, preventive care was utilized by our study population. Encouraging preventive care and addressing the multifactorial causes of caries – particularly oral hygiene and nutrition – should be integrated into public health programming tailored for displaced populations.

In a study among Afghan students at Kabul University of Medical Sciences, women were more likely to express concern about and demonstrate preventive behaviors for maintaining oral health compared to males [19]. This distinction by sex is consistent across Eastern Mediterranean and Central Asian countries [20, 21], as well as in the United States [22]. These studies, however, reflect populations living in comparatively stable settings, potentially indicating better access to both knowledge and care provisions. The health status and needs of refugees and asylum seekers are context-specific, often resulting in unique profiles both within and between displaced communities, as described by Pavlopoulou et al., (2017) among recent arrivals of children in Greece [12]. In a cross-sectional study of displaced persons in informal settlements in Northern Greece who were utilizing oral health services, over 70% of study participants were male [23]. Males in our study had more oral health consultations than females and were more likely to have preventive consultations. This could be due to a range of factors, including the higher proportion of males among the displaced populations captured in our study or changes in health seeking behaviors among either men or women when displaced from their countries of origin.

While we did not assess knowledge and intent, utilization patterns can offer important insights into access and behaviors related to oral health. The deviation from expected sex-specific patterns for displaced populations in Greece could be attributable, in part, to the unique demographic characteristics of our study population. Immediately prior to our study period, the number of asylum applications from unaccompanied minors throughout Europe reached record highs, approaching 90,000 applications [24]. Two-thirds of these applications were from unaccompanied or separated Afghan and Syrian children, as reflected by our study population. Future research should target approaches to the unique and site-specific demographic profiles of displaced populations and investigate knowledge and behaviors around oral health in these contexts.

### Dental consultations and comorbid conditions

Because of the importance of oral healthcare – for a complaint, a procedure, or prevention – as an indicator of overall health and wellbeing, as well as timely and appropriate care, we explored the relationship between any dental consultation and general health consultations for upper respiratory tract infections (URTIs) and reproductive health concerns among women of reproductive age.

Viruses and bacteria causing upper respiratory tract infections (URTIs) can exacerbate and be exacerbated by poor oral health [4]. URTIs are highly prevalent in refugee and displaced communities [25–27]. Our findings reflect the negatively reinforcing relationship between URTIs and oral health. However, in a study in Hong Kong, children who received dental care for decayed, missing, or filled teeth were less likely to have URTIs [28]. The authors of this study noted that the findings were contrary to expected findings and highlighted the need to further investigate the role that oral health care plays in both adaptive and innate immune response to pathogens in the oral cavity. Given the nascent state of the mechanistic understanding of the modulating role of oral microbiota with respiratory infections underscores the importance of continuing to characterize these relationships. With these considerations in mind, the role of oral health should be included in public health planning and programming as a means of complementing environmental interventions to address URTI risk in complex settings.

The importance of oral health in pregnancy is widely acknowledged [29], but oral health promotion and treatment are not adequately incorporated by primary care providers into pre and peri-natal care [30]. While the connection between pregnancy and oral health is most frequently highlighted, changes in hormone levels throughout the life course also significantly influence oral health for females [31, 32]. In our study, girls and women with consultations for reproductive health concerns were more likely to have any dental consultation or consultations for caries specifically. This finding reinforces the importance of promoting and integrating oral health and hygiene throughout the life course into primary and reproductive care.

### Comparing dental consultations among Afghans and Syrians

Individuals from Afghanistan and Syria – the most prevalent countries of origin in our study population – consistently have significant oral health challenges. In multiple studies of school-aged children in Herat, Afghanistan, preventative and restorative care for caries and other dental conditions were inadequate to meet population needs [33, 34]. There were high prevalences of caries and periodontal disease among displaced Syrian refugees in Za’atari refugee camp in Jordan [35, 36] and among Syrian children receiving dental screenings in Istanbul, Türkiye [37]. There is not substantial evidence pointing to differences in oral health needs by country of origin, although the importance of considering country of origin and associated circumstances surrounding displacement when assessing population needs [12, 38, 39]. Additional research is needed to provide an evidence-base for developing dental health interventions and education among these populations, considering their specific cultural and situational contexts.

### Limitations

Data only represented individuals with consultations at Médecins du Monde clinics, so baselines and demographic information for entire camp populations were not available. The study population was assumed to be a reasonably representative sample of total camp populations as well as a reasonable proxy for refugees and asylum seekers in the Eastern Mediterranean from 2016 – 2017. This assumption has implications for both the interpretation and generalizability of findings. The demographic profile of the study population is reflective of United Nations High Commissioner for Refugees and European Union migration tracking, and the high prevalence of oral health conditions is consistent with other studies from similar populations and settings. Clinical data were recorded by providers from a range of linguistic and cultural backgrounds, and standard diagnostic codes were not used. Accordingly, we could have misclassified reasons for consultations due to differences in documenting diagnoses, treatment, and related notes. As noted throughout the discussion, small cell counts for comorbidities in the context of specific dental conditions could have contributed to deviations from expected patterns. However, given that the data used in this study include individuals from the 12 MdM dental clinics operated across mainland Greece, these findings still provide important context for the situational profile of oral health needs among displaced persons in the Eastern Mediterranean.

## Conclusions

The Eastern Mediterranean has become a critical transit route for refugees and asylum seekers from African, Central and Southeast Asian, and Eastern Mediterranean countries, with more than 2.7 million crossings into the European Union via this route from January 2014 through January 2024 [40]. Dental health is bidirectionally related to overall health and wellbeing and must be seen as part of an essential package of health services available to refugees and asylum seekers. Future research should continue to explore the increasingly important relationship between oral and systemic health. Further, Salim and Tawari (2021), noted that language barriers and competing essential interests, including but not limited to the need to address both legal and financial concerns, contributed to underutilization of dental health services among displaced persons globally [41]. Considering whole-of-person approaches in asylum and immigration reform could contribute to increased healthcare – including dental care – access for displaced populations [42]. Given the protracted migration emergency in the Eastern Mediterranean, expanded analyses are needed to identify continuing needs and inform potential public health programming to address the oral health needs of and subsequent care provided for displaced populations.

## Supplementary Information

Supplementary Material 1.

## Data Availability

The datasets used and/or analyzed during the current study are available from the corresponding author upon reasonable request.

## Abbreviations

CI: Confidence interval
MdM: Médecins du Monde
OR: Odds ratio
PM: Proportional morbidity
SADDD: Sex, age, and disability disaggregated data
UNDRR: United Nations Office for Disaster Risk Reduction
URTI: Upper respiratory tract infection
