# Supplementary material for: Oral health among refugees and asylum seekers utilizing Médecins du Monde clinics in mainland Greece, 2016–2017
Source: BMC Oral Health. 2024 Sep 6;24:1045. doi: 10.1186/s12903-024-04841-2 (PMC11378550; doi:10.1186/s12903-024-04841-2)
Supplement: Supplementary file 1 — Supplementary Material 1. [file 12903_2024_4841_MOESM1_ESM.docx]

**Figure 2.** Odds ratios with 95% confidence intervals comparing reasons for consultations between Afghans and Syrians.


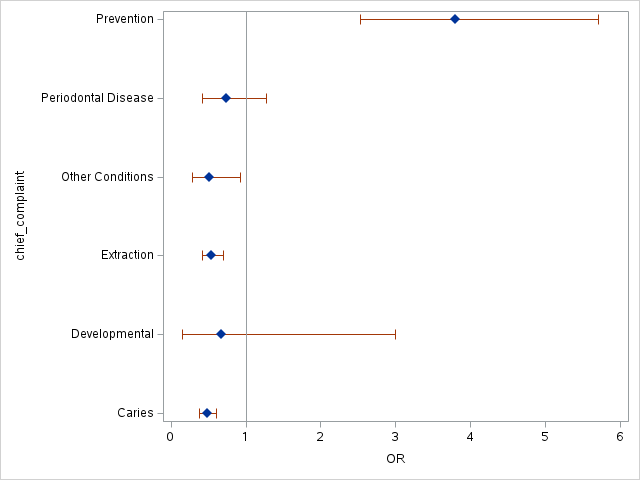


Dental Condition
